# Diabetes-Driven Retinal Neurodegeneration: Its Role in the Pathogenesis of Diabetic Retinopathy

**DOI:** 10.3390/biomedicines13061328

**Published:** 2025-05-29

**Authors:** Ilaria Viganò, Silvia Galbiati, Emanuela Aragona, Daniela Gabellini, Rosangela Lattanzio, Vittoria Pedon, Giulia Basile, Alessandro Arrigo, Francesco Bandello, Gianpaolo Zerbini

**Affiliations:** 1 Complications of Diabetes Unit, Diabetes Research Institute, IRCCS San Raffaele Scientific Institute, 20132 Milan, Italy; vigano.ilaria@hsr.it (I.V.); galbiati.silvia@hsr.it (S.G.); gabellini.daniela@hsr.it (D.G.); 2 Department of Ophthalmology, IRCCS San Raffaele Scientific Institute, 20132 Milan, Italy; aragona.emanuela@hsr.it (E.A.); lattanzio.rosangela@hsr.it (R.L.); v.pedon@studenti.unisr.it (V.P.); giuliabasile95@gmail.com (G.B.); arrigo.alessandro@hsr.it (A.A.); bandello.francesco@hsr.it (F.B.); 3 Eye Repair Unit, Division of Neuroscience, IRCCS San Raffaele Scientific Institute, 20132 Milan, Italy

**Keywords:** diabetic retinopathy, neurodegeneration, prevention

## Abstract

Diabetic retinopathy is a complication of diabetes characterized by an extremely low rate of progression. It takes several years to move from the onset of diabetes, both type 1 and type 2, to the development of retinal microaneurysms, then leading to proliferative diabetic retinopathy and vision loss. The recent demonstration that retinal microaneurysms are preceded and, possibly, caused by a subclinical neurodegeneration mainly affecting the neurovascular unit has suggested, on one hand, the possible existence of a previously unknown early neurodegenerative stage of diabetic retinopathy and, on the other, that an early “neuroprotective” treatment could end up preventing the development of the microvascular stages. This review summarizes the present situation in the field and focuses on the prevention of diabetic retinopathy, which seems, for the first time, to be within reach.

## 1. Introduction

There is presently still no cure for diabetes. Both type 1 and type 2 diabetes are successfully treated with insulin [1] or with oral hypoglycemic agents [2], but the slight difference between cure and treatment, when protracted for years or decades, may still result in the development of diabetic complications [3,4].

Diabetic retinopathy (DR), neuropathy and nephropathy are defined as the microvascular complications of diabetes [3,5]. Just like neuropathy and nephropathy, DR is specific to patients affected by diabetes, but it is not mandatory; there are, in fact, individuals, so-called medalists, with 50 or more years of diabetes who are free of complications [6,7].

Microangiopathy represents the key feature of DR [8], characterized by abnormal growth and increased vascular permeability of retinal micro-vessels. The parallel death of pericytes worsens the situation and increases the permeability, thus leading to retinal edema. Dysfunctional vascular regeneration coupled to inflammation, oxidative stress and hyperosmolar stress [9] cooperate to accelerate the progression rate of the complication.

**Figure 1:**
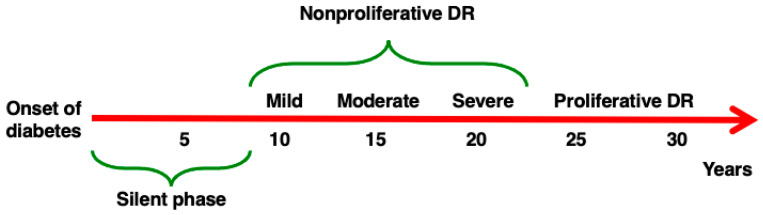
Progression of DR. DR may develop in patients affected by both Type 1 and Type 2 diabetes. After an initial silent phase, the four stages of DR develop in sequence. (1) Mild non-proliferative (NP) DR (characterized by the development of microaneurysms), (2) moderate NPDR (abnormal structure and caliber of blood vessels), (3) severe NPDR (reduced perfusion, diffuse ischemia), (4) proliferative DR (proliferation of new fragile blood vessels). The rate of progression of DR described here is approximative, as the natural history of the complication differs in different individuals.

DR is characterized by an extremely slow rate of progression [10], as described in Figure 1, but all attempts thus far to stop or prevent its evolution have been substantially unsuccessful, and the only effective treatments for advanced DR currently are laser photocoagulation and intravitreal anti-VEGF injections [11,12].

The recent demonstration that microaneurysms might be preceded by a subclinical retinal neurodegeneration [13,14] could, on one hand, help clarify the pathogenesis of DR [15] and, on the other, allow us to set up, for the first time, a successful preventive strategy for DR.

## 2. Diabetic Retinopathy

DR is a well-established complication of diabetes that affects both type 1 and type 2 diabetic patients [16,17]. After the onset of diabetes, it takes a few years to develop retinal microaneurysms, the first clinical sign of DR. This stage, also known as non-proliferative retinopathy, may be followed by the development of intra-retinal hemorrhages, macular edema, hard exudates and capillary dropout resulting in microinfarcts resulting in diffuse ischemia. In a subset of patients, retinal ischemia leads to an angiogenic switch characterized by the increased synthesis of local vascular endothelial growth factor (VEGF), causing rapid growth of new, incompetent and leaky capillaries that can easily break and cause major bleeding and consequent hemovitreous. All the clinical signs described above are referable to a diffuse disease affecting small vessels and capillaries of the retina, thus justifying the inclusion of DR among the microvascular complications of diabetes [16,17].

From an epidemiological point of view, and despite the recent significant improvements in the therapeutic approaches for both types of diabetes, proliferative DR presently remains a prominent cause of blindness in working-age individuals in developed countries [18,19] and a cause of major psychological burden in the entire diabetic population [20].

## 3. Neurovascular Unit

Distribution and structure of the vasculature in the different tissues of an organism have always been supposed to be homogeneous. In 1885, however, Paul Ehrlich discovered that, after injecting a water-soluble dye into the circulatory system of different animals, the tracer stained all the organs with the exception of the brain, retina and spinal cord. A few years later a student of Paul Ehrlich, Edwin Goldman, showed that the phenomenon was reversible because, when the dye was injected inside the cerebrospinal fluid, it was able to stain the brain, but the rest of the organism remained unaffected by the procedure [21]. The brain and retina are therefore separated from the rest of the organism, and this was the first demonstration of the existence of a Blood-Brain Barrier (BBB) and of a Blood–Retina Barrier (BRB) [21,22].

**Figure 2:**
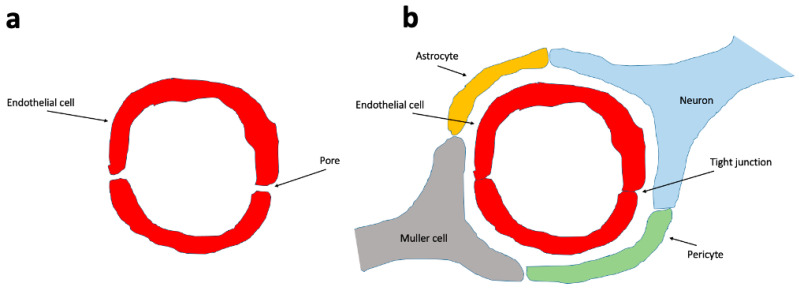
The blood vessels that do not form a neurovascular unit have a more permeable endothelium. (**a**) The endothelial cells of all vessels have tight junctions. The non-neurovascular unit blood vessels’ endothelial cells do have numerous inter-endothelial cell gaps (permeable pores), making them more permeable to certain molecules. In the neurovascular unit (**b**), the endothelial cells are connected to each other by tight junctions and lack permeable pores. Endothelial cells are surrounded by pericytes, neurons and astrocytes that reinforce and stabilize the neurovascular unit.

The BRB consists of the inner and outer BRB. The inner BRB (iBRB) consists of retinal capillary endothelial cells that form tight junctions and give rise to the microvascular component. This component, in addition to pericytes, neural cells such as ganglion cells, amacrine cells, bipolar and horizontal cells, immune cells (including microglia and macrophages), and macroglia (Muller cells), constitutes the retinal neurovascular unit (NVU, Figure 2), which also exists in the brain with the presence of BBB [22,23].

The other retinal barrier, the oBRB, stands between the choriocapillaris and the retina and is formed by the choroid, Bruch’s membrane and the retinal pigmented epithelium.

Breakdown of the BRB that may follow the disruption of tight junctions and scattered neovascularization, but also neurodegeneration and pericyte dropout, results in cellular stress, retinal ischemia, leakage, and, finally, the development of diffuse edema. A scenario that is common to different retinal diseases [22,23].

## 4. Diseases Characterized by Retinal Neurodegeneration

The retina consists of a combination of vascular and neuronal components. When the light signal reaches the retina, it crosses all the neuronal layers and goes straight to the outer (deeper) side of the retina, where photoreceptors are situated.

**Figure 3:**
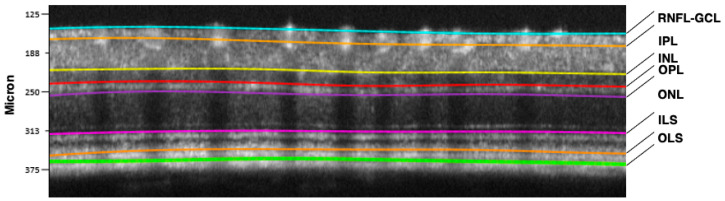
Retinal segmentation performed by the Micron IV OCT System and InSight software, Version 1. (Phoenix Technology Group, Pleasanton, CA, USA) in a C57BL6J mouse. RNFL-GCL: retinal nerve fiber layer—ganglion cell layer complex (containing axons and bodies of retinal ganglion cells); IPL: inner plexiform layer (synaptic connections between amacrine, bipolar, and retinal ganglion cells); INL: inner nuclear layer (cell bodies of amacrine, bipolar, and horizontal cells); OPL: outer plexiform layer (synaptic connections between photoreceptors, bipolar and horizontal cells); ONL: outer nuclear layer (cell bodies of photoreceptors, rods and cones); ILS: photoreceptor inner segments; OLS: photoreceptor outer segments.

Starting from there, the signal is progressively sent back through the vertical pathway formed by photoreceptors, bipolar and retinal ganglion cells (RGCs) with their regulation by amacrine and horizontal neurons (horizontal pathway). RGC axons form the optic nerve, the “bridge” between the retina and the brain cortex [24,25]. A representative optical coherence tomography (OCT) scan of a normal murine retina describing in detail the different neuronal layers is shown in Figure 3.

This system is extremely complex and is actually the target of a number of neurodegenerative diseases [26].

Glaucoma: Behind the term glaucoma lies a number of different optic neuropathies sharing the same clinical feature: the progressive degeneration of RGC and the lesion of the optic nerve. The disease is often associated with an increased intraocular pressure, but this is not always the case, as approximately half of the patients have a normal intraocular pressure. The strongest risk factors for glaucoma are age, followed by intraocular pressure and family history of the disease. Genetic factors are also involved in the predisposition to develop glaucoma [27,28].

Age-related Macular Degeneration (AMD): In contrast with the wet form of AMD, which is the consequence of neovascularization, the dry form (the most common, 80% of the cases) is characterized by the suffering and dysfunction of retinal pigmented epithelium, by the progressive loss of photoreceptors, and by the degeneration of the retina. In both forms of AMD, the presence of drusen (fragments of cellular waste material) becomes detectable inside the retina. Risk factors for AMD are age, smoking habits, family history of the disease, dyslipidemia and hypertension [29,30].

Retinitis Pigmentosa: An inherited disease with a slow progression, characterized by the progressive death of photoreceptors and atrophy of retinal pigmented epithelium, eventually leading to blindness. The pathogenesis of the disease is the consequence of mutations in a number of specific genes of photoreceptors (in particular rods), causing degeneration of cones and dysfunction of retinal pigment epithelium and RGC [31,32].

The above-described diseases have the retina as the major target, but retinal neurodegeneration may be a “side” manifestation of brain diseases, as described below.

Alzheimer’s disease: Plaques of amyloid beta and neurofibrils of tau protein (the classic markers of Alzheimer’s disease in the brain) have been demonstrated in the retina of patients affected by Alzheimer’s disease, suggesting that these dysfunctions could explain the pathogenesis of Alzheimer’s-associated retinal degeneration, a phenomenon characterized, in particular, by thinning of the retinal nerve fiber layer and loss of RGC [33,34].

Parkinson’s disease: Thinning of the retinal nerve fiber layer, of the ganglion cell-inner plexiform layer and of the retina in total characterizes the eye of the patients affected by Parkinson’s disease, confirming that retinal neurodegeneration is a central feature of the disease [35,36,37].

## 5. Diabetes-Driven Retinal Neurodegeneration

The concept that diabetes can directly induce the degeneration and death of neurons is a relatively new one. Endothelial cells have always been considered as the specific target of diabetes and, in particular, of glucotoxicity [38,39].

Peripheral nerves have also been identified as a target of diabetes, and in fact, once affected, they are directly involved in the pathogenesis of diabetic neuropathy [40]. Neurons, also known to express the insulin receptor, have been considered resistant to the dysmetabolism induced by diabetes [41,42].

We now know that diabetes may cause retinal neurodegeneration in different ways. Hyperglycemia by itself may damage retinal vessels and neural cells through multiple mechanisms, such as overproduction of advanced glycation end-products (AGEs) and activation of the polyol, hexosamine and diacylglycerol-protein kinase C pathways. In particular, increased production of reactive oxygen species (ROS) in the Krebs cycle may act as a “unifying mechanism” of the above pathways, as suggested by Brownlee et al. [43,44].

Recent evidence suggests that, in the case of diabetes, inflammation plays a major role in the early loss of RGC, the phenomenon that is now considered by many authors as the “primum movens” in the pathogenesis of DR [45,46]. Among the different transcription factors involved in the development of inflammation, the nuclear factor kappa-light-chain-enhancer of activated B cells (NF-kB) seems to have a specific role in the case of diabetes [47]. NF-kB is a widely expressed inducible transcription factor, a key regulator of the activity of many genes involved in inflammation and immune responses, and a major player in the modulation of cellular proliferation and apoptosis.

NF-kB, once activated, induces the transcription of pro-inflammatory proteins (such as inducible nitric oxide synthase (iNOS), intercellular adhesion molecule-1 (ICAM), and cytokines). Diabetes was shown to activate NF-kB in murine retinas [48,49] and to cause the nuclear relocation of its p65 subunit inside retinal endothelial cells, pericytes, RGCs, and cells of the inner nuclear layer [50,51]. DNA-binding activity of NF-kB is increased in retinal endothelial cells and pericytes exposed to elevated glucose concentrations. Finally, an increased NF-kB expression was demonstrated in epiretinal membranes of patients with proliferative DR [52]. Altogether these findings confirm the central role of NF-kB in the pathogenesis of early stages of DR.

From a functional/histologic point of view, features of diabetes-induced neurodegeneration, primarily attributable to metabolic alterations [53], include the activation of glial cells [54], the reduction in retinal neuronal function and the apoptosis of neuronal cells (in particular amacrine cells, RGC and photoreceptors) [15,55]. The search for biomarkers of retinal neurodegeneration of clinical use is presently under way; circulating biomarkers of increasing interest are glial-fibrillary acidic protein (GFAP) and neurofilament light chain (NFL) [56]. Recently, CXC-motif chemokine ligand 13 (CXCL13) has also been added to the list of candidate biomarkers [57].

As described above, the retina, just like the brain, is characterized by the presence of the neurovascular unit (NVU), a structure that is based on the physical and biochemical interaction between neurons, glial cells and retinal vessels [58]. The reciprocal interactions between the various components of the NVU may be significantly modified in the case of diabetes that causes the unbalancing of the mediators contained in the NVU itself, such as ATP, lactate, nitric oxide, arachidonic acid and other lipids [59]. Several studies have clearly demonstrated that diabetes-driven metabolic alterations cause neuronal degeneration, as evidenced by the impairment of glutamatergic and dopaminergic neurotransmitter signaling [60], by the modification of the dendritic fields [61] and by the reduced expression of synaptic proteins [58] induced by chronic hyperglycemia.

Another consequence of prolonged hyperglycemia is the activation of glycogen synthase kinase-3beta, an event that induces hyperphosphorylation of the tau protein and down-regulation of beta-catenin. These dysfunctions end up leading to mitochondrial abnormalities of RGC and to an acceleration of apoptosis [62].

In parallel, neuronal damage may also follow the development of insulin resistance and the reduction in sensitivity to neurotrophic factors, such as brain-derived neurotrophic factor (BDNF) [16].

Neurons by themselves may undergo apoptosis due to the persistent and uncontrolled hyperglycemia [63]. Glial cells may also endure significant modifications in case of diabetes, as demonstrated by the dysfunctional interconversion of glutamate and glutamine [64], the altered regulation of potassium channels [65], and the activation of glutamate-aspartate transporter and intermediate filament proteins, such as the glial fibrillary acidic protein (GFAP). Astrocytes, which are in tight contact with retinal blood vessels and with synapses [59], may also be dysfunctional, for still poorly understood reasons, in the case of diabetes [66]. Microglia probably play an adaptive role, at least in the short period, but may contribute to retinal damage when chronically stimulated [67].

In humans, the hypothesis of early neuronal damage induced by diabetes is supported by electrophysiology, imaging and clinical evidence, which demonstrate alterations already in the early stages of DR [68,69,70,71,72]. Electrophysiological alterations are assessed by the technique of multifocal electroretinography (mfERG), which allows us to highlight that, in the case of diabetes, the timing of mfERG is delayed in retinal areas affected by vascular lesions and that the delay increases with the increase in the dimensions of the lesions. The amplitude of mfERG is instead normal or slightly reduced both inside and outside the retinal lesions [71,73,74].

Anatomical distribution of neuro-retinal alterations can be precisely investigated by optical coherence tomography (OCT), which, in the early stages of the disease, is able to demonstrate a significant thinning of the retinal nerve fiber layer and photoreceptor layers in patients with type 1 diabetes, while in type 2 diabetic patients, there is a diffuse thinning of the retinal nerve fiber layer, ganglion cell layer, inner plexiform layer, inner nuclear layer, outer plexiform layer and photoreceptor layers [75]. In the early phase of DR, during which no alterations are noted on fundus examination, a reduced contrast sensitivity and an alteration in color vision have been demonstrated [76,77,78].

## 6. Diabetes-Driven Retinal Neurodegeneration as the First Stage of Diabetic Retinopathy

DR is a complication of both type 1 and type 2 diabetes characterized by an extremely slow rate of progression. It takes years, in most cases decades, to move from the onset of diabetes to the development of retinal microaneurysms, the “classic” first signs of the complication [16,17]. Even though it has always been reasonable to assume that diabetes should actually start acting on the retina since its first manifestation, we have been, until recently, unable to document morphological changes in a diabetic retina during the silent phase that precedes the appearance of microaneurysms.

Even though neurodegeneration was for the first time suggested to be involved in the pathogenesis of DR in 1962 by JM Bloodworth [79], it took 40 more years before it was shown that retinal neurodegeneration may precede the vascular stage of DR and therefore that, at least potentially, it could also contribute to its development [80,81,82]. Neuronal dysfunction involves different cells inside the NVU, and typical signs of neurodegeneration, such as glial activation and RGC death, have been identified in animal models of diabetes and ex vivo in retinas of diabetic patients before any detectable signs of vascular stages of DR [63,83,84,85,86,87,88].

During the last few years, the technology applied to the field of ophthalmology has significantly improved, and new techniques have been developed and subsequently applied to clarify the role of early retinal neurodegeneration in the pathogenesis of DR [89,90,91,92]. OCT [13,93], OCT angiography (OCTA) [68,94], flicker-evoked retinal vasodilation [95,96] and mfERG [97,98], have rapidly become of common use, first as research tools (both in humans and in different animal models) and then rapidly translated into clinical practice.

The results of these studies substantially confirmed that, in the case of diabetes, retinal neurodegeneration precedes or parallels the development of the vascular signs of DR. As a consequence of these findings, in 2017 the American Diabetes Association turned the definition of DR from “vascular complication of diabetes” to “neurovascular complication of diabetes” [10].

The issue that still remains to be clarified is whether retinal neurodegeneration is a necessary or an optional step in the pathogenesis of DR. The evidence that patients with long-lasting type 1 diabetes and absence of DR are also free of retinal neurodegeneration [99] is in line with the hypothesis that the absence of neurodegeneration may protect from the subsequent development of DR. Conversely, the results of the large EUROCONDOR study [98] performed in type 2 diabetic patients suggest that, in a subset of patients, DR develops without a preceding retinal neurodegeneration.

A possible alternative way to clarify the role of retinal neurodegeneration in the pathogenesis of DR consists of verifying whether it is possible to avoid the development of the vascular stages of DR through an early preventive treatment based on agents known to have a neuroprotective effect.

## 7. Diabetes-Driven Retinal Neurodegeneration as a Pharmacological Target to Prevent the Vascular Stages of Diabetic Retinopathy

As described above, retinal neurodegeneration plays a significant role in the pathogenesis of DR, and it has been observed to precede microvascular alterations [100,101]. Early retinal neurodegeneration therefore represents the ideal target when planning to set up a pharmacologic strategy aimed to stop the progression of the complication and to avoid the development of its sight-threatening vascular stages. A number of different approaches have been implemented in the last few years to reach this aim.

Fenofibrate is an oral medication of the fibrate class and a peroxisome proliferator-activated receptor alpha (PPARalfa) agonist [102]. Two major clinical trials (Fenofibrate Intervention and Event Lowering in Diabetes (FIELD) Study and Action to Control Cardiovascular Risk in Diabetes (ACCORD) Eye Study) have demonstrated that fenofibrate is able to interrupt the progression of DR in patients affected by type 2 diabetes [103,104,105]. Studies on a murine model of type 2 diabetes (db/db mice) have revealed that fenofibrate influences circulating lipids, modulates gliosis and improves electroretinogram (ERG) abnormalities [106], thus confirming its neuroprotective (by reducing reactive gliosis, apoptosis and glutamate excitotoxicity [103]) and vasculotropic effect [107].

Calcium dobesilate (CaD) is a drug that has been approved for the treatment of DR in different countries, but only recently has its neuroprotective effect been deeply studied, thus confirming its antioxidant and anti-inflammatory properties and its vascular effects [108]. Bogdanov et al. demonstrated that CaD is able to determine a decrease in oxidative stress and a downregulation of several pro-inflammatory cytokines, like IL-6, KC analog IL-8, TNF-alpha and MCP-1, determining a reduction in both neurodegeneration and vascular leakage in a murine model of diabetes (db/db mice) [109]. Additionally, it has been shown that CaD reduces the levels of endothelin-1 (ET-1), a vasoconstrictor peptide, thereby decreasing the activity of endothelin B receptor (ETB-R) expressed by retinal neurons and endothelin A receptor (ETA-R) expressed by blood vessels [110].

Bosentan is a dual ETA-R and ETB-R antagonist [111]. Upregulation of ET-1 and ETB-R was demonstrated in the retinas of diabetic patients [112]. Bosentan, given via topical administration (eye drops), was able to reduce the levels of retinal ET-1, ETB-R and ETA-R (after quantification of immunofluorescence) in db/db mice, thus resulting in a reduction in both reactive gliosis and apoptosis rates [112], in an improvement of the integrity of the vasculature, and in a reduction in the number of acellular capillaries, a characteristic of DR common to diabetic patients and animal models of diabetes [113].

TNF-alpha blockers, such as Etanercept and CNT05048, are drugs targeting TNF-alpha, a well-established inflammatory cytokine [54]. TNF-alpha activates the NF-kB pathway, thus inducing iNOS expression, ROS formation and upregulation of adhesion molecules such as VCAM, ICAM-1 and E-selectin, all of which contribute to leukostasis and DR progression [114]. The blockade of TNF-alpha inflammatory pathway results in apoptosis reduction, anti-VEGF effect and decrease in leukostasis, confirming the central role of inflammation in the pathogenesis of DR and underlying its potential as a pharmacological target [115]. Clinical translation of these results is expected when clinical trials are completed [116].

Suppressors of cytokine signaling (SOCS) are a family of intracellular proteins whose role is to inhibit cytokine signaling through the downregulation of the JAK/signal transducer and activator of transcription (STAT) signaling pathway in different cell types. In particular, SOCS1 and SOCS3 modulate the innate and adaptive immune response [117]. Treatment based on cell-penetrating peptides derived from the kinase inhibitory region of SOCS1 and SOCS3 (R9-SOCS1-KIR and R9-SOCS3-KIR) was shown to reduce inflammation, oxidative stress and neo-angiogenesis, pathways involved in retinal neurodegeneration and vascular alterations, thus confirming their possible role in managing DR [118]. R9-SOCS3-KIR was shown to reduce inflammation and oxidative stress in a human retinal pigment epithelium cell line [119].

Non-steroidal anti-inflammatory drugs (NSAIDs) are a widely prescribed pharmacological class, known for its anti-inflammatory, antipyretic and analgesic effects [54]. Yukiko et al. demonstrated that administration of Sulindac has the capability to halt DR progression through the inhibition of cyclooxygenase (COX)-1 and COX-2 and through the blockade of the NF-kB pathway [120]. Other NSAIDs, such as indomethacin, bromfenac and nepafenac, were shown to be effective in reducing inflammation, ameliorating alterations in retinal thickness and in arteriole diameter [121]. In a recent study, a polypill, including metformin, aspirin, simvastatin, and angiotensin-converting enzyme inhibitors, was administered in a mixed murine model of Alzheimer’s disease and type 2 diabetes, showing promising results in ameliorating metabolic parameters, cognitive impairment, brain atrophy, neuronal and synaptic loss, amyloid burden, tau phosphorylation, central inflammation and central spontaneous bleeding [122]. Common pathways relating dementia and DR have been identified, suggesting new therapeutic opportunities to prevent the diabetic complications [55]. However, more studies on the application of NSAIDs in diabetes-driven retinal neurodegeneration are needed to verify its neuroprotective effects.

Antioxidant active compounds have been demonstrated to be effective in reducing inflammation and oxidative stress, both playing a pivotal role in DR pathogenesis, as confirmed in vitro and in animal studies [123]. Oxidative stress determines an increase in ROS production, thereby determining vascular damage via capillary cell apoptosis and inflammation and neurodegeneration in the early phases of DR [124]. For all these reasons, antioxidant molecules could represent a valid tool to counteract retinal neurodegeneration, in particular when administered in the early phase of the disease [125].

Peptides with neurotrophic and anti-angiogenic activities, such as pigment epithelial growth factor (PEDF), somatostatin, glucagon-like peptide 1 (GLP-1) or molecules that prevent the degradation of these peptides, such as DPP-IV inhibitors, were studied in experimental models and were shown to be effective strategies in slowing down DR progression [126,127,128,129]. Sitagliptin (DPP-IV inhibitor) administered as eye drops improved the dysfunction of the neurovascular unit in the Trpv2+/− rat (a non-diabetic animal model that develops DR-like lesions), determining a significant reduction in retinal thinning, glial activation, inflammation, oxidative stress, abnormal vasodilatation and vasodegeneration, thus confirming its protective effect against the development of retinal neurodegeneration [130]. Moreover, sitagliptin and liraglutide (a GLP-1 receptor agonist) were shown to be effective in reducing the phosphorylation of tau, a toxic mediator of retinal ganglion cell synaptic neurodegeneration, underlying the important role of correct synaptic connectivity in order to prevent retinal degeneration in DR [131]. Other molecules, like taurine [132] and lutein [133], may also have a conservative role, protecting in particular the retinal synaptic connections.

Complement modulators have also been taken into consideration as a possible pharmacological strategy to prevent retinal neurodegeneration [131]. Innate immunity is upregulated in DM, determining chronic inflammation and, ultimately, neuronal and vascular damage [134]. However, the role of the activation of the complement system in the pathogenesis of diabetes-driven retinal neurodegeneration remains unclear [135].

Neurotrophins, including nerve growth factor (NGF) and brain-derived neurotrophic factor (BDNF), were studied to assess the possibility of preventing retinal neurodegeneration in DR and, consequently, to avert retinal vascular damage [136,137]. Studies on a murine model of diabetes (Ins2akita mouse) have demonstrated that topical administration of NGF has the capability of preventing early retinal neurodegeneration and subsequent loss of retinal pericytes and development of acellular capillaries, two retinal dysfunctions of DR common to humans and animal models of diabetes [138]. A key point in this field is to identify a way to perform a long-lasting administration of neurotrophins directly in the eye to avoid multiple eye drop deliveries and to prevent the systemic side effects of these powerful growth factors [137]. As a possible solution, Pelusi et al. demonstrated the effectiveness of bioengineered human corneal lenticule as a promising delivery system for recombinant human NGF (rhNGF) [139].

Mesenchymal stromal/stem cells (MSCs) have also been explored as a potential therapy in DR. MSCs are known to release factors such as fibroblast growth factor (bFGF), VEGF, insulin-like growth factor (IGF), NGF and BDNF, factors that are known to protect from neurodegeneration [140]. In particular, a study based on human retinal progenitor cells (RPCs) derived from fetal retinas, embryonic stem cells (ESCs) and induced pluripotent stem cells (iPSCs) demonstrated the capacity of these cells to integrate into the retina of a murine model of diabetes when injected under the retina [141]. In this way it is possible to prevent neuronal loss and diabetes-driven retinal neurodegeneration [141]. In addition, amniotic stem cells, placental stem cells, neural stem cells and human umbilical cord mesenchymal stem cells (HUCMSCs) were all found effective in regenerating neuronal cells in murine models of diabetes [142]. A list of neuroprotective drugs is described in Table 1.

## 8. Conclusions and Perspectives

There is a substantial agreement in the recent literature that diabetes, besides retinal microangiopathy, is also the cause of the early development of retinal neurodegeneration.

Retinal neurodegeneration is a well-known common feature of diseases such as glaucoma, AMD, Retinitis pigmentosa, Alzheimer’s disease and Parkinson’s disease. In all these cases, however, behind the development of retinal dysfunction, there is familial and/or genetic predisposition. Conversely, in the case of DR, retinal neurodegeneration seems to be the direct consequence of the dysmetabolism induced by diabetes and, in particular, of high ambient glucose.

Whether retinal neurodegeneration may also represent the initial cause of DR is presently still unclear, even though a large number of basic and clinical studies are aimed at clarifying this issue. The possibility that early retinal neurodegeneration may represent a necessary step toward the development of the vascular stages in some, but not in all, diabetic patients has also been suggested [13].

Whatever the case, the discovery that diabetes, by itself, may cause retinal neurodegeneration and that this dysfunction could, at least potentially, contribute to the development of the subsequent stages of DR paves the way toward the search for specific treatments that can prevent or treat the dysfunction. As described above, a large number of basic and clinical studies are presently ongoing with the aim to identify the best possible neuroprotective agent able to prevent the retinal neurodegeneration induced by diabetes.

## Figures and Tables

**Table 1:** List of drugs used in the prevention and/or treatment of diabetes-driven retinal neurodegeneration.

Name of Drugs	Properties
Fenofibrate	Neuroprotective and vasculotropic effect: reduction in inflammation, oxidative stress and anti-apoptotic activity [102,103,104,105,106,107]
Calcium dobesilate	Neuroprotective and vasculotropic effect: reduction in inflammation, oxidative stress, anti-apoptotic activity, and blockade of ET-1, ETA-R and ETB-R [108,109,110]
Endothelin receptor blockers	Neuroprotective (ETB-R) and vasculotropic (ETA-R) effects [111,112,113]
TNF-alpha blockers	Anti-inflammatory and neuroprotective effect [54,114,115,116]
SOCS proteins	Neuroprotective and vasculotropic effect: inhibition of neuroinflammation and vascular leakage [117,118,119]
Non-steroidal anti-inflammatory drugs (NSAIDs)	Anti-inflammatory and neuroprotective effect [54,120,121,122]
Antioxidants	Neuroprotective and vasculoprotective effect [123,124,125]
Peptides with neurotrophic and anti-angiogenic properties: PEDF, GLP-1, GLP-1 receptor agonists, DPP-IV inhibitors, somatostatin	Neuroprotective and vasculotropic effect. Activity on synaptic connectivity (DPP-IV inhibitors and GLP-1 receptor agonists) [126,127,128,129,130,131]
Taurine and lutein	Improvement of synaptic connections [132,133]
Modulators of complement activation	Protect from early synaptic loss and dendritic atrophy induced by complement cascade activation [131,134,135]
Neurotrophins, nerve growth factor (NGF) and brain-derived neurotrophic factor (BDNF)	Neuroprotective, neurotrophic and vasculoprotective effects [136,137,138,139]
Stem cell-based treatments	Neuroprotective effect exerted either by the secretion of specific growth factors (FGF, VEGF, IGF, NGF, BDNF) or through the direct integration into the retina [140,141,142]
